# (*meso*-5,5,7,12,12,14-Hexamethyl-1,4,8,11-tetra­azacyclo­tetra­deca­ne)copper(II) bis­(*O*,*S*-dibenzyl dithio­phosphate)

**DOI:** 10.1107/S1600536809030037

**Published:** 2009-08-08

**Authors:** Jian-Shen Feng, Li-Ke Zou, Bin Xie, Yu Wu

**Affiliations:** a College of Chemistry & Pharmaceutical Engineering, Sichuan University of Science & Engineering, Zigong 643000, People’s Republic of China

## Abstract

In the crystal structure of the title compound, [Cu(C_16_H_36_N_4_)](C_14_H_14_O_2_PS_2_)_2_, the Cu^II^ atom is located on an inversion center and is chelated by four N atoms of the macrocyclic *meso*-5,5,7,12,12,14- hexa­methyl-1,4,8,11-tetra­azacyclo­tetra­decane ligand in a square-planar geometry, with Cu—N distances of 2.013 (3) and 2.014 (3) Å. In the crystal structure, one *O*,*S*-dibenzyl dithio­phosphate counter-anion links with the Cu^II^ complex cation through N—H⋯O and N—H⋯S hydrogen bonding. During the synthesis, the structure of the anion re-arranged from *O*,*O*′-dibenzyl dithio­phosphate in the starting material to *O*,*S*-dibenzyl dithio­phosphate in the title compound.

## Related literature

For a related Ni^II^ complex, see: Xie *et al.* (2008 ▶). For bond-length data, see Allen *et al.* (1987 ▶).
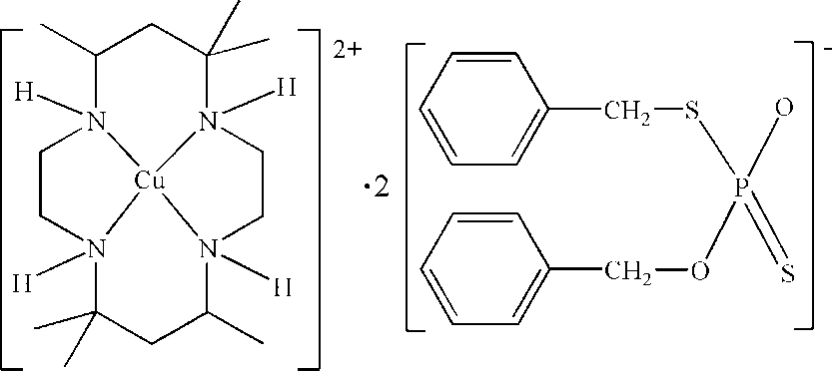


## Experimental

### 

#### Crystal data

[Cu(C_16_H_36_N_4_)](C_14_H_14_O_2_PS_2_)_2_
*M* *_r_* = 966.71
Monoclinic, 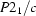

*a* = 11.476 (4) Å
*b* = 17.592 (4) Å
*c* = 11.945 (4) Å
β = 99.78 (2)°
*V* = 2376.4 (13) Å^3^
*Z* = 2
Mo *K*α radiation
μ = 0.75 mm^−1^
*T* = 289 K
0.44 × 0.40 × 0.35 mm

#### Data collection

Enraf–Nonius CAD-4 diffractometer
Absorption correction: ψ scan (North *et al.*, 1968 ▶) *T*_min_ = 0.730, *T*_max_ = 0.770
4797 measured reflections
4420 independent reflections
2900 reflections with *I* > 2σ(*I*)
*R*_int_ = 0.006
3 standard reflections every 300 reflections intensity decay: 6.7%

#### Refinement

*R*[*F* ^2^ > 2σ(*F* ^2^)] = 0.051
*wR*(*F* ^2^) = 0.154
*S* = 1.04
4420 reflections
275 parameters
H-atom parameters constrained
Δρ_max_ = 0.45 e Å^−3^
Δρ_min_ = −0.69 e Å^−3^

### 

Data collection: *CAD-4 Software* (Enraf–Nonius, 1989 ▶); cell refinement: *CAD-4 Software*; data reduction: *XCAD4* (Harms & Wocadlo, 1995 ▶); program(s) used to solve structure: *SHELXS97* (Sheldrick, 2008 ▶); program(s) used to refine structure: *SHELXL97* (Sheldrick, 2008 ▶); molecular graphics: *ORTEP-3 for Windows* (Farrugia, 1997 ▶); software used to prepare material for publication: *SHELXL97*.

## Supplementary Material

Crystal structure: contains datablocks I, global. DOI: 10.1107/S1600536809030037/xu2568sup1.cif

Structure factors: contains datablocks I. DOI: 10.1107/S1600536809030037/xu2568Isup2.hkl

Additional supplementary materials:  crystallographic information; 3D view; checkCIF report

## Figures and Tables

**Table 1:** Hydrogen-bond geometry (Å, °)

*D*—H⋯*A*	*D*—H	H⋯*A*	*D*⋯*A*	*D*—H⋯*A*
N1—H1⋯S1	0.91	2.61	3.359 (3)	140
N2—H2⋯O2^i^	0.91	1.85	2.762 (4)	176

Symmetry code: (i) 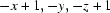.

**Fig. 1.**
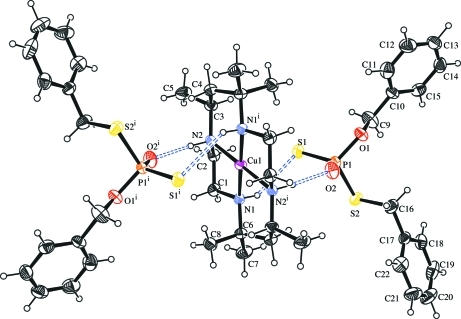
A view of the title compound showing the atom-labelling scheme. Displacement ellipsoids are drawn at the 30% probability level. Hydrogen-bonds are shown as dashed lines. H atoms are represented as small spheres of arbitrary radii. [Symmetry code: (i) -x + 1, -y, -z + 1].

**Fig. 2.**
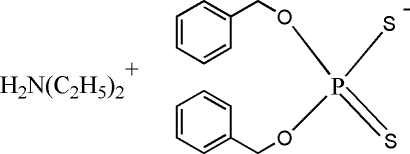
The starting material [NH2(C2H5)2]+[(PhCH2O)2PS2]-.

## supplementary crystallographic information

### Comment

As part of an investigation to the tetramine macrocyclic transition metal complexs and their potential applications as artificial enzyme models, we have reported the structures of [Ni(tet-a)][S_2_P(OCH_2_Ph)_2_]_2_, where tet-a is a macrocyclic tetramine, *meso*-5,5,7,12,12,14- hexamethyl-1,4,8,11-tetraazacyclotetradecane (Xie *et al.*, 2008). Here we report the crystal structure of the corresponding title Cu^II^ compound, [Cu(tet-a)][OSP(OCH_2_Ph)(SCH_2_Ph)]_2_.

In the title crystal structure, the complex cation [Cu(tet-a)]^2+^ possesses square-planar geometry about the Cu^II^ atom (Fig. 1), which lies across a centre of inversion and is four-coordinated by four N atoms of the tetramine macrocyclic ligand tet-a. All the bond lengths and angles in the adduct are generally within normal ranges (Allen *et al.*, 1987). The two *O*,*S*-dibenzyl dithiophosphate anions act as counter-ions to balance the charge of the Cu^II^ complex cation, they interact with the complex cation through N—H···O and N—H···S hydrogen bonds (Table 1).

### Experimental

A solution of *meso*-5,5,7,12,12,14- hexamethyl-1,4,8,11-tetraazacyclotetradecane dihydrate (0.32 g, 1 mmol) and CuCl_2_.2H_2_O (0.17 g, 1 mmol) in 20 ml methanol was added to a solution of diethylammonium *O*,*O*'-dibenzyldithiophosphate, [NH_2_(C_2_H_5_)_2_]^+^[(PhCH_2_O)_2_PS_2_]^-^ (Fig. 2), (0.77 g, 2 mmol) in 20 ml methanol. The mixture was refluxed for 24 h, then cooled to room temperature, the dark-violet precipitate was collected by filtration, washed with small amounts of methanol. A solution of the title compound in DMSO was kept at room temperature, and dark-violet block crystals suitable for X-ray diffraction studies were obtained in three months.

It should be noted that the title compound contains an unexpected re-arrangement product of the anion; in the starting reagent, [NH_2_(C_2_H_5_)_2_]^+^[(PhCH_2_O)_2_PS_2_]^-^, both of the two benzyl-groups bonded with O atoms, but in the title compound one of them migrated to the neighbouring S atom.

### Refinement

All H atoms attached to C and N atom were fixed geometrically and treated as riding with C—H = 0.98 Å (methine), 0.97 Å (methylene), 0.96 Å (methyl) or 0.93 Å (aromatic) and N—H = 0.91 Å with *U*_iso_(H) = 1.2*U*_eq_(C, N) or *U*_iso_(H) = 1.5*U*_eq_(methyl).

### Crystal data

**Table:**

[Cu(C_16_H_36_N_4_)](C_14_H_14_O_2_PS_2_)_2_	*F*(000) = 1022
*M**_r_* = 966.71	*D*_x_ = 1.351 Mg m^−^^3^
Monoclinic, *P*2_1_/*c*	Mo *K*α radiation, λ = 0.71073 Å
Hall symbol: -P 2ybc	Cell parameters from 29 reflections
*a* = 11.476 (4) Å	θ = 4.4–11.5°
*b* = 17.592 (4) Å	µ = 0.75 mm^−^^1^
*c* = 11.945 (4) Å	*T* = 289 K
β = 99.78 (2)°	Block, dark-violet
*V* = 2376.4 (13) Å^3^	0.44 × 0.40 × 0.35 mm
*Z* = 2	

### Data collection

**Table:**

Enraf–Nonius CAD-4 diffractometer	2900 reflections with *I* > 2σ(*I*)
Radiation source: fine-focus sealed tube	*R*_int_ = 0.006
graphite	θ_max_ = 25.6°, θ_min_ = 1.8°
ω/2θ scans	*h* = −13→13
Absorption correction: ψ scan (North *et al.*, 1968)	*k* = 0→21
*T*_min_ = 0.730, *T*_max_ = 0.770	*l* = −4→14
4797 measured reflections	3 standard reflections every 300 reflections
4420 independent reflections	intensity decay: 6.7%

### Refinement

**Table:**

Refinement on *F*^2^	Primary atom site location: structure-invariant direct methods
Least-squares matrix: full	Secondary atom site location: difference Fourier map
*R*[*F*^2^ > 2σ(*F*^2^)] = 0.051	Hydrogen site location: inferred from neighbouring sites
*wR*(*F*^2^) = 0.154	H-atom parameters constrained
*S* = 1.04	*w* = 1/[σ^2^(*F*_o_^2^) + (0.0968*P*)^2^] where *P* = (*F*_o_^2^ + 2*F*_c_^2^)/3
4420 reflections	(Δ/σ)_max_ < 0.001
275 parameters	Δρ_max_ = 0.45 e Å^−^^3^
0 restraints	Δρ_min_ = −0.69 e Å^−^^3^

### Special details

**Table:**

Geometry. All e.s.d.'s (except the e.s.d. in the dihedral angle between two l.s. planes) are estimated using the full covariance matrix. The cell e.s.d.'s are taken into account individually in the estimation of e.s.d.'s in distances, angles and torsion angles; correlations between e.s.d.'s in cell parameters are only used when they are defined by crystal symmetry. An approximate (isotropic) treatment of cell e.s.d.'s is used for estimating e.s.d.'s involving l.s. planes.
Refinement. Refinement of *F*^2^ against ALL reflections. The weighted *R*-factor *wR* and goodness of fit *S* are based on *F*^2^, conventional *R*-factors *R* are based on *F*, with *F* set to zero for negative *F*^2^. The threshold expression of *F*^2^ > σ(*F*^2^) is used only for calculating *R*-factors(gt) *etc*. and is not relevant to the choice of reflections for refinement. *R*-factors based on *F*^2^ are statistically about twice as large as those based on *F*, and *R*- factors based on ALL data will be even larger.

### Fractional atomic coordinates and isotropic or equivalent isotropic displacement parameters (Å^2^)

**Table:**

	*x*	*y*	*z*	*U*_iso_*/*U*_eq_	
Cu1	0.5000	0.0000	0.5000	0.0373 (2)	
S1	0.45680 (10)	0.16085 (8)	0.65146 (9)	0.0633 (3)	
S2	0.60091 (9)	0.16270 (6)	0.90486 (8)	0.0523 (3)	
P1	0.46223 (8)	0.11046 (6)	0.79879 (8)	0.0426 (3)	
O1	0.3474 (2)	0.12698 (17)	0.8538 (2)	0.0550 (7)	
O2	0.4729 (3)	0.02701 (17)	0.8069 (3)	0.0610 (8)	
N1	0.6456 (2)	0.06574 (17)	0.5164 (2)	0.0376 (7)	
H1	0.6323	0.1022	0.5669	0.045*	
N2	0.4441 (2)	0.05885 (17)	0.3564 (2)	0.0369 (7)	
H2	0.4682	0.0312	0.3002	0.044*	
C1	0.6406 (3)	0.1077 (2)	0.4085 (3)	0.0475 (9)	
H1A	0.6712	0.0762	0.3535	0.057*	
H1B	0.6886	0.1533	0.4211	0.057*	
C2	0.5149 (3)	0.1285 (2)	0.3647 (3)	0.0431 (9)	
H2A	0.4867	0.1642	0.4159	0.052*	
H2B	0.5086	0.1522	0.2906	0.052*	
C3	0.3165 (3)	0.0731 (2)	0.3165 (3)	0.0444 (9)	
H3	0.2894	0.1101	0.3679	0.053*	
C4	0.2455 (3)	0.0014 (2)	0.3192 (3)	0.0480 (9)	
H4A	0.2786	−0.0366	0.2747	0.058*	
H4B	0.1658	0.0117	0.2804	0.058*	
C5	0.2935 (4)	0.1062 (3)	0.1966 (4)	0.0708 (14)	
H5A	0.3397	0.1514	0.1943	0.106*	
H5B	0.2111	0.1183	0.1757	0.106*	
H5C	0.3152	0.0695	0.1442	0.106*	
C6	0.7638 (3)	0.0344 (2)	0.5661 (3)	0.0415 (8)	
C7	0.8558 (4)	0.0984 (3)	0.5855 (4)	0.0667 (13)	
H7A	0.8587	0.1237	0.5149	0.100*	
H7B	0.9321	0.0773	0.6147	0.100*	
H7C	0.8343	0.1342	0.6392	0.100*	
C8	0.8018 (4)	−0.0231 (2)	0.4853 (4)	0.0546 (10)	
H8A	0.7366	−0.0559	0.4572	0.082*	
H8B	0.8662	−0.0528	0.5246	0.082*	
H8C	0.8268	0.0029	0.4228	0.082*	
C9	0.3028 (5)	0.2003 (3)	0.8600 (5)	0.0731 (14)	
H9A	0.2887	0.2232	0.7851	0.088*	
H9B	0.3597	0.2314	0.9092	0.088*	
C10	0.1885 (3)	0.1965 (2)	0.9066 (3)	0.0476 (9)	
C11	0.1010 (5)	0.1453 (3)	0.8683 (4)	0.0669 (12)	
H11	0.1119	0.1113	0.8114	0.080*	
C12	−0.0002 (4)	0.1428 (3)	0.9107 (5)	0.0750 (14)	
H12	−0.0577	0.1070	0.8832	0.090*	
C13	−0.0188 (4)	0.1915 (3)	0.9925 (4)	0.0703 (14)	
H13	−0.0894	0.1895	1.0208	0.084*	
C14	0.0652 (5)	0.2437 (3)	1.0342 (4)	0.0700 (14)	
H14	0.0530	0.2772	1.0914	0.084*	
C15	0.1705 (4)	0.2460 (2)	0.9893 (4)	0.0579 (11)	
H15	0.2284	0.2817	1.0163	0.070*	
C16	0.5930 (4)	0.1139 (3)	1.0376 (3)	0.0676 (14)	
H16A	0.5285	0.1346	1.0710	0.081*	
H16B	0.5778	0.0603	1.0230	0.081*	
C17	0.7064 (3)	0.1235 (3)	1.1178 (3)	0.0492 (10)	
C18	0.7318 (4)	0.1904 (3)	1.1759 (4)	0.0618 (12)	
H18	0.6778	0.2303	1.1655	0.074*	
C19	0.8376 (6)	0.1983 (3)	1.2498 (4)	0.0812 (17)	
H19	0.8536	0.2437	1.2893	0.097*	
C20	0.9167 (5)	0.1428 (4)	1.2658 (4)	0.0847 (18)	
H20	0.9882	0.1496	1.3145	0.102*	
C21	0.8919 (5)	0.0752 (3)	1.2098 (5)	0.0809 (16)	
H21	0.9456	0.0352	1.2222	0.097*	
C22	0.7873 (4)	0.0666 (3)	1.1349 (4)	0.0663 (12)	
H22	0.7718	0.0211	1.0955	0.080*	

### Atomic displacement parameters (Å^2^)

**Table:**

	*U*^11^	*U*^22^	*U*^33^	*U*^12^	*U*^13^	*U*^23^
Cu1	0.0326 (3)	0.0406 (4)	0.0394 (3)	0.0051 (3)	0.0081 (2)	0.0141 (3)
S1	0.0623 (7)	0.0895 (9)	0.0385 (5)	0.0165 (6)	0.0100 (5)	0.0071 (5)
S2	0.0512 (6)	0.0614 (7)	0.0420 (5)	−0.0119 (5)	0.0015 (4)	0.0061 (5)
P1	0.0407 (5)	0.0505 (6)	0.0381 (5)	0.0035 (4)	0.0110 (4)	−0.0066 (4)
O1	0.0499 (16)	0.0567 (18)	0.0628 (17)	0.0093 (14)	0.0223 (13)	−0.0028 (14)
O2	0.083 (2)	0.0394 (16)	0.0649 (18)	0.0009 (15)	0.0241 (16)	−0.0094 (14)
N1	0.0390 (15)	0.0383 (17)	0.0360 (15)	0.0010 (13)	0.0080 (12)	0.0041 (13)
N2	0.0401 (15)	0.0398 (17)	0.0317 (14)	0.0087 (13)	0.0085 (12)	0.0053 (13)
C1	0.051 (2)	0.049 (2)	0.044 (2)	−0.0070 (18)	0.0104 (17)	0.0137 (17)
C2	0.054 (2)	0.034 (2)	0.0422 (19)	0.0028 (17)	0.0115 (16)	0.0112 (16)
C3	0.040 (2)	0.045 (2)	0.047 (2)	0.0107 (17)	0.0050 (16)	0.0115 (17)
C4	0.041 (2)	0.056 (2)	0.045 (2)	0.0057 (18)	−0.0001 (16)	0.0022 (18)
C5	0.060 (3)	0.088 (4)	0.059 (3)	0.010 (3)	−0.003 (2)	0.036 (3)
C6	0.0346 (18)	0.043 (2)	0.046 (2)	−0.0012 (16)	0.0029 (15)	0.0039 (17)
C7	0.052 (3)	0.067 (3)	0.075 (3)	−0.019 (2)	−0.004 (2)	0.010 (2)
C8	0.053 (2)	0.058 (3)	0.055 (2)	0.011 (2)	0.0157 (19)	0.006 (2)
C9	0.077 (3)	0.051 (3)	0.100 (4)	0.000 (2)	0.041 (3)	0.006 (3)
C10	0.040 (2)	0.050 (2)	0.056 (2)	0.0059 (18)	0.0167 (17)	0.0112 (19)
C11	0.084 (3)	0.055 (3)	0.064 (3)	0.006 (3)	0.015 (2)	−0.004 (2)
C12	0.056 (3)	0.080 (4)	0.087 (4)	−0.015 (3)	0.007 (3)	0.006 (3)
C13	0.051 (3)	0.096 (4)	0.068 (3)	0.015 (3)	0.019 (2)	0.028 (3)
C14	0.081 (3)	0.082 (4)	0.049 (2)	0.035 (3)	0.015 (2)	0.003 (2)
C15	0.057 (3)	0.050 (3)	0.062 (3)	0.002 (2)	−0.003 (2)	0.001 (2)
C16	0.055 (3)	0.102 (4)	0.045 (2)	−0.023 (3)	0.0031 (19)	0.018 (2)
C17	0.046 (2)	0.063 (3)	0.039 (2)	−0.013 (2)	0.0062 (16)	0.0060 (19)
C18	0.074 (3)	0.066 (3)	0.048 (2)	−0.006 (2)	0.018 (2)	0.005 (2)
C19	0.114 (5)	0.084 (4)	0.044 (3)	−0.044 (4)	0.008 (3)	−0.004 (3)
C20	0.070 (3)	0.113 (5)	0.061 (3)	−0.036 (3)	−0.016 (3)	0.023 (3)
C21	0.059 (3)	0.093 (4)	0.085 (4)	0.012 (3)	−0.004 (3)	0.029 (3)
C22	0.072 (3)	0.055 (3)	0.070 (3)	−0.010 (2)	0.008 (2)	0.004 (2)

### Geometric parameters (Å, °)

**Table:**

Cu1—N2^i^	2.013 (3)	C7—H7B	0.9600
Cu1—N2	2.013 (3)	C7—H7C	0.9600
Cu1—N1^i^	2.014 (3)	C8—H8A	0.9600
Cu1—N1	2.014 (3)	C8—H8B	0.9600
S1—P1	1.9619 (15)	C8—H8C	0.9600
S2—C16	1.818 (4)	C9—C10	1.510 (6)
S2—P1	2.0729 (15)	C9—H9A	0.9700
P1—O2	1.475 (3)	C9—H9B	0.9700
P1—O1	1.596 (3)	C10—C15	1.359 (6)
O1—C9	1.394 (5)	C10—C11	1.368 (6)
N1—C1	1.478 (4)	C11—C12	1.344 (7)
N1—C6	1.491 (4)	C11—H11	0.9300
N1—H1	0.9100	C12—C13	1.343 (7)
N2—C2	1.465 (5)	C12—H12	0.9300
N2—C3	1.482 (4)	C13—C14	1.364 (7)
N2—H2	0.9100	C13—H13	0.9300
C1—C2	1.493 (5)	C14—C15	1.404 (7)
C1—H1A	0.9700	C14—H14	0.9300
C1—H1B	0.9700	C15—H15	0.9300
C2—H2A	0.9700	C16—C17	1.489 (5)
C2—H2B	0.9700	C16—H16A	0.9700
C3—C4	1.506 (5)	C16—H16B	0.9700
C3—C5	1.527 (5)	C17—C22	1.356 (6)
C3—H3	0.9800	C17—C18	1.372 (6)
C4—C6^i^	1.528 (5)	C18—C19	1.382 (7)
C4—H4A	0.9700	C18—H18	0.9300
C4—H4B	0.9700	C19—C20	1.325 (8)
C5—H5A	0.9600	C19—H19	0.9300
C5—H5B	0.9600	C20—C21	1.370 (8)
C5—H5C	0.9600	C20—H20	0.9300
C6—C8	1.513 (5)	C21—C22	1.379 (7)
C6—C4^i^	1.528 (5)	C21—H21	0.9300
C6—C7	1.533 (5)	C22—H22	0.9300
C7—H7A	0.9600		
			
N2^i^—Cu1—N2	180.0	C4^i^—C6—C7	108.6 (3)
N2^i^—Cu1—N1^i^	85.80 (11)	C6—C7—H7A	109.5
N2—Cu1—N1^i^	94.20 (12)	C6—C7—H7B	109.5
N2^i^—Cu1—N1	94.20 (12)	H7A—C7—H7B	109.5
N2—Cu1—N1	85.80 (11)	C6—C7—H7C	109.5
N1^i^—Cu1—N1	180.0	H7A—C7—H7C	109.5
C16—S2—P1	100.18 (15)	H7B—C7—H7C	109.5
O2—P1—O1	102.66 (17)	C6—C8—H8A	109.5
O2—P1—S1	119.89 (13)	C6—C8—H8B	109.5
O1—P1—S1	112.60 (12)	H8A—C8—H8B	109.5
O2—P1—S2	110.77 (14)	C6—C8—H8C	109.5
O1—P1—S2	105.65 (12)	H8A—C8—H8C	109.5
S1—P1—S2	104.60 (7)	H8B—C8—H8C	109.5
C9—O1—P1	121.8 (3)	O1—C9—C10	109.2 (4)
C1—N1—C6	115.4 (3)	O1—C9—H9A	109.8
C1—N1—Cu1	107.0 (2)	C10—C9—H9A	109.8
C6—N1—Cu1	120.7 (2)	O1—C9—H9B	109.8
C1—N1—H1	103.9	C10—C9—H9B	109.8
C6—N1—H1	103.9	H9A—C9—H9B	108.3
Cu1—N1—H1	103.9	C15—C10—C11	118.1 (4)
C2—N2—C3	112.7 (3)	C15—C10—C9	119.2 (4)
C2—N2—Cu1	106.3 (2)	C11—C10—C9	122.6 (4)
C3—N2—Cu1	121.0 (2)	C12—C11—C10	121.9 (5)
C2—N2—H2	105.2	C12—C11—H11	119.1
C3—N2—H2	105.2	C10—C11—H11	119.1
Cu1—N2—H2	105.2	C13—C12—C11	120.5 (5)
N1—C1—C2	108.7 (3)	C13—C12—H12	119.8
N1—C1—H1A	110.0	C11—C12—H12	119.8
C2—C1—H1A	110.0	C12—C13—C14	120.4 (4)
N1—C1—H1B	110.0	C12—C13—H13	119.8
C2—C1—H1B	110.0	C14—C13—H13	119.8
H1A—C1—H1B	108.3	C13—C14—C15	118.7 (4)
N2—C2—C1	108.1 (3)	C13—C14—H14	120.7
N2—C2—H2A	110.1	C15—C14—H14	120.7
C1—C2—H2A	110.1	C10—C15—C14	120.4 (4)
N2—C2—H2B	110.1	C10—C15—H15	119.8
C1—C2—H2B	110.1	C14—C15—H15	119.8
H2A—C2—H2B	108.4	C17—C16—S2	109.9 (3)
N2—C3—C4	111.1 (3)	C17—C16—H16A	109.7
N2—C3—C5	111.7 (3)	S2—C16—H16A	109.7
C4—C3—C5	109.3 (3)	C17—C16—H16B	109.7
N2—C3—H3	108.2	S2—C16—H16B	109.7
C4—C3—H3	108.2	H16A—C16—H16B	108.2
C5—C3—H3	108.2	C22—C17—C18	118.4 (4)
C3—C4—C6^i^	119.0 (3)	C22—C17—C16	120.9 (4)
C3—C4—H4A	107.6	C18—C17—C16	120.6 (4)
C6^i^—C4—H4A	107.6	C17—C18—C19	119.8 (5)
C3—C4—H4B	107.6	C17—C18—H18	120.1
C6^i^—C4—H4B	107.6	C19—C18—H18	120.1
H4A—C4—H4B	107.0	C20—C19—C18	121.6 (5)
C3—C5—H5A	109.5	C20—C19—H19	119.2
C3—C5—H5B	109.5	C18—C19—H19	119.2
H5A—C5—H5B	109.5	C19—C20—C21	119.3 (5)
C3—C5—H5C	109.5	C19—C20—H20	120.3
H5A—C5—H5C	109.5	C21—C20—H20	120.3
H5B—C5—H5C	109.5	C20—C21—C22	119.8 (5)
N1—C6—C8	109.6 (3)	C20—C21—H21	120.1
N1—C6—C4^i^	108.1 (3)	C22—C21—H21	120.1
C8—C6—C4^i^	111.6 (3)	C17—C22—C21	121.0 (5)
N1—C6—C7	110.2 (3)	C17—C22—H22	119.5
C8—C6—C7	108.8 (3)	C21—C22—H22	119.5

Symmetry codes: (i) −*x*+1, −*y*, −*z*+1.

### Hydrogen-bond geometry (Å, °)

**Table:**

*D*—H···*A*	*D*—H	H···*A*	*D*···*A*	*D*—H···*A*
N1—H1···S1	0.91	2.61	3.359 (3)	140
N2—H2···O2^i^	0.91	1.85	2.762 (4)	176

Symmetry codes: (i) −*x*+1, −*y*, −*z*+1.
